# Spatial Distribution of Stony Desertification and Key Influencing Factors on Different Sampling Scales in Small Karst Watersheds

**DOI:** 10.3390/ijerph15040743

**Published:** 2018-04-13

**Authors:** Zhenming Zhang, Yunchao Zhou, Shijie Wang, Xianfei Huang

**Affiliations:** 1 Forest Resource and Environment Research Center of Guizhou Province, Guizhou University, Guiyang 550025, China; zhang6653579@163.com (Z.Z.); hxfswjs@gznu.edu.cn (X.H.); 2 College of Forestry, Guizhou University, Guiyang 550025, China; 3 Puding Karst Ecosystem Research Station of Guizhou Province, Puding 562100, China; wangshijie@vip.skleg.cn; 4 State Key Laboratory of Environmental Geochemistry, Institute of Geochemistry, Chinese Academy of Science, Guiyang 550002, China

**Keywords:** different sampling scales, spatial distribution, stony desertification characteristics, karst, small watershed

## Abstract

Karst areas are typical ecologically fragile areas, and stony desertification has become the most serious ecological and economic problems in these areas worldwide as well as a source of disasters and poverty. A reasonable sampling scale is of great importance for research on soil science in karst areas. In this paper, the spatial distribution of stony desertification characteristics and its influencing factors in karst areas are studied at different sampling scales using a grid sampling method based on geographic information system (GIS) technology and geo-statistics. The rock exposure obtained through sampling over a 150 m × 150 m grid in the Houzhai River Basin was utilized as the original data, and five grid scales (300 m × 300 m, 450 m × 450 m, 600 m × 600 m, 750 m × 750 m, and 900 m × 900 m) were used as the subsample sets. The results show that the rock exposure does not vary substantially from one sampling scale to another, while the average values of the five subsamples all fluctuate around the average value of the entire set. As the sampling scale increases, the maximum value and the average value of the rock exposure gradually decrease, and there is a gradual increase in the coefficient of variability. At the scale of 150 m × 150 m, the areas of minor stony desertification, medium stony desertification, and major stony desertification in the Houzhai River Basin are 7.81 km^2^, 4.50 km^2^, and 1.87 km^2^, respectively. The spatial variability of stony desertification at small scales is influenced by many factors, and the variability at medium scales is jointly influenced by gradient, rock content, and rock exposure. At large scales, the spatial variability of stony desertification is mainly influenced by soil thickness and rock content.

## 1. Introduction

Soil is a continuum with uneven changes, and soil properties present obvious spatial variability [1]. The foundation of soil science research is to obtain detailed and accurate spatial distribution information on soil properties [2]. The sampling scale has a decisive influence on the acquisition accuracy and quantitative expression of soil properties and spatial variability information [3]. Theoretically, the narrower the sampling scale, the lower the prediction error of an interpolation. If sampling is oversized, it is difficult to guarantee interpolation accuracy [4]. However, excessively high sampling density will require more manpower, material resources, financial resources and long work cycles [5]. How to determine a reasonable sampling density during regional research on soil science is a key and difficult point of the present research on soil science [6]. Different sampling scales lead to different characteristics, including different influencing factors and different evolution mechanisms and processes [7]. Therefore, the key influencing factors can be reliably revealed only if the sampling scale is in line with the intrinsic scale of the phenomenon to be studied. In karst areas, the earth surface is greatly uneven, the landscape is fragmented, and the key influencing factors vary with sampling scale.

The concept of stony desertification was first proposed in the early 1980s, and later, it was defined as a term representing the process of the transition from vegetation-covered and soil-covered karst areas to karst landscapes covered by bare rocks [8]. Karst areas in China are typical ecologically fragile areas, and the problem of stony desertification has become the most serious ecological and economic problem as well as a source of disasters and poverty in the area [9]. According to the experience in treating typical stony desertification areas, the treatment requires guidance on the driving mechanisms and theories at different spatial scales [10]. For example, a great number of studies regarding soil organic carbon at different scales and in different landforms have been conducted over the past several decades [11]. However, the results of these studies differ significantly from each other. For instance, the reported global soil organic carbon storage ranged from 504 Pg to 3000 Pg with a mean value of 1460.5 Pg. One of the main reasons for this variation is the high spatial variability in the organic carbon content of soils, which is caused by various landforms and complicated changing geological conditions [12]. In particular, it is necessary to determine the positive and negative impacts of natural and human effects on the progress of stony desertification as well as their respective contribution rates [13]. Therefore, the distribution characteristics of stony desertification and their relations with environmental factors on different sampling scales are of great importance for the understanding of the progress of a karst ecosystem and have received widespread attention [14]. At present, the majority of studies on the spatial distribution of stony desertification are limited to single factors and the spatial correlation [15,16]. There are very few comprehensive and systematic quantitative analyses of the impacts of various factors on the spatial distribution of stony desertification, and there are even fewer studies on problems about differences in scale. In this paper, GIS and geo-statistics are combined to reveal patterns of the spatial distribution characteristics of stony desertification at different sampling scales. Multiple stepwise regression and Pearson correlation analysis are used to explore the key influencing factors of stony desertification characteristics on non-sampling scales. These methods are aimed at revealing the determinants of the spatial distribution of stony desertification and the differences among different scales, and the results provide references for the treatment of stony desertification in karst areas.

## 2. Materials and Methods

### 2.1. Study Area

The study region (105°40′43″–105°48′2″ E, 26°12′29″–26°17′15″ N) is located in Puding County in the central part of Guizhou Province in southwestern China [17], including the three towns of Chengguan (CG), Maguan (MG) and Baiyan (BY), and it covers an area of 75 km^2^. The elevation is between 1223.4 and 1567.4 m.a.s.l., and the air pressure is between 806.1 and 883.8 hpa. There are three major categories of soil: limestone soil, paddy soil and yellow soil. A total of 92.78% of the available flatlands (excluding construction land and watersheds) is used for cropland, and only 3.49% of flatlands are left as various forestland and grassland. The other 3.73% of flatlands consist of uncultivated land. A total of 42.13% of lands on mountains consist of cropland, and 44.95% of lands on mountains are left for various forestland and grassland. A total of 12.95% of lands on mountains consist of uncultivated land due to severe environmental conditions and poor geographical characteristics. The vegetation mainly includes *Cupressus funebris* Endl., *Populus adenopoda* Maxim, *Toona sinensis* (A. Juss.) Roem., *Pyrus pyrifolia* Burm Nakai., *Catalpa bungei* C. A. Mey., *Pinus massoniana* Lamb., and *Pyracantha fortuneana* (Maxim.) Li. The main crops are *Oryzasativa Oryzaglaberrima*, *Zea mays* Linn. Sp., *Glycine max* (Linn.) Merr, and *Helianthus annuus*. There are 7 soil types in the study area: Xan Udic Fernalisols, Black Lithomorphic Isohumisols, Cab Udi Orthic Entisols, Cab High-fertility Orthic Anthrosols, Cab Low-fertility Orthic Anthrosols, Cab Medium-fertility Orthic Anthrosols, and Fec Hydragric Anthrosols.

### 2.2. Data Source

**Figure 1:**
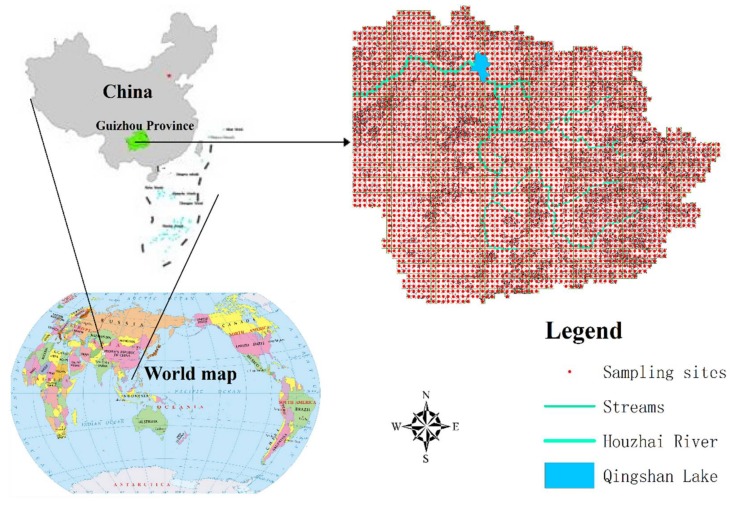
The location of Houzhai River basin and the distribution of sample sites.

Sampling plots were designed with a grid-based sampling method, and there was a total of 2755 sampling grids (150 m × 150 m) consisting of 22,057 soil samples. The sampling depth is 100 cm. The sampling sites were defined as the center of each sampling grid (Figure 1). The soil samples were air dried, ground and prepared for analysis as required by the laboratory. Rock exposures were measured by linear interception using tape in the field. Rock exposure was assessed by the average percentage of rock occupied in 10 m distance, and the texts were repeated for four times in east, west, north and south direction, respectively.

### 2.3. Classification of Different Grid Scales

**Figure 2:**
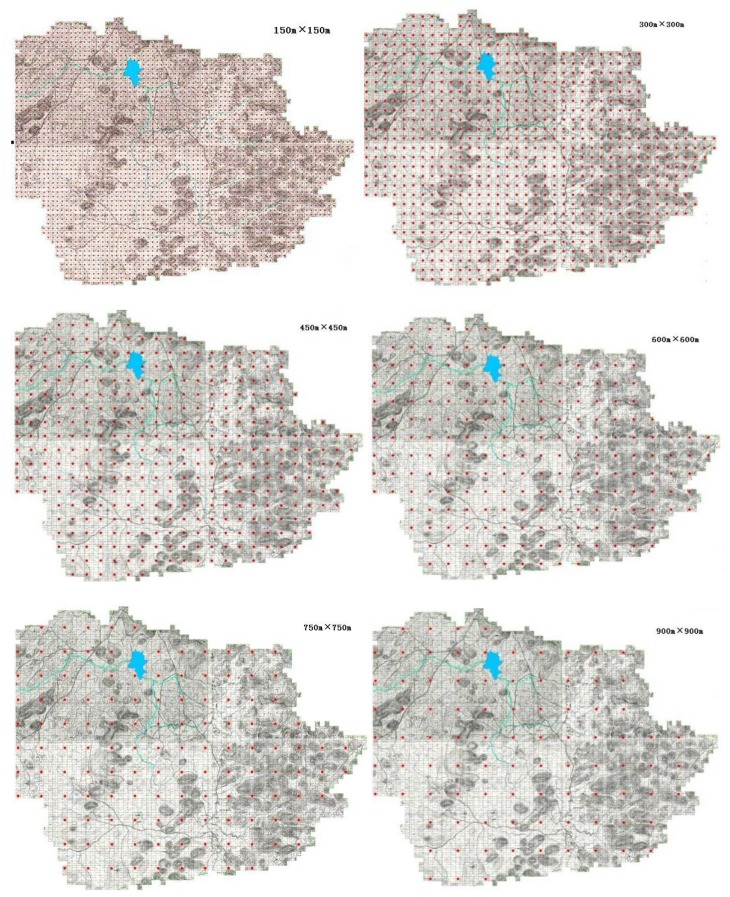
Plots of soil sample distribution under different sampling scales.

The grid scale determines the density and sampling point data. To study the impacts of different sampling scales on the revelation of spatial variability of soil organic carbon, ArcGIS software is adopted to extract cells from the 2755 grids at 150 m × 150 m to obtain 802 grids of 300 m × 300 m; then, these grids were classified into six grid scales: 150 m × 150 m, 300 m × 300 m, 450 m × 450 m, 600 m × 600 m, 750 m × 750 m and 900 m × 900 m (Figure 2). At the same time, to ensure the reliability of the conclusions and reduce the unreliability resulting from repeated sampling, subsets of five samples were selected without replacement.

### 2.4. Calculations and Statistical Analysis

Classical statistical methods can be used to describe some overall characteristics of the coverage of rock exposures (CRE), but they are unable to characterize its spatial variability. Therefore, this study used a geostatistical method to quantify the constitutive properties and randomness of the CRE to analyze the patterns of spatial variation in the CRE more accurately [18].

A semivariance function (*h*) was used to describe the spatial heterogeneity of the soil properties. The semivariance function was used to obtain the variation in the semivariance function value with an increase in the distance of the sample; the scatter plots were fitted with a Gaussian model and other theoretical models. When the soil properties met a two-order stationary assumption and the intrinsic hypothesis and when the sample size was large enough, the semivariance theory variation function (*h*) formula was used. The semivariance (r(h)) is as follows [19]:

(1) $$r(h)=\frac{1}{2N(h)}\sum_{i=1}^{N(h)}{\left[Z(xi)-Z(xi+h)\right]}^{2},$$

where *Z* is the measured soil property, *x* is the sample location, and *N(h)* is the number of pairs of locations separated by a lag distance *h*. The semivariogram expresses the relationship between the semivariance and the lag distance (*h*). The semivariogram typically increases from a value at *h* = 0 (identified as the nugget) to a maximum value (identified as the sill), which is created using ArcGIS software. The rock exposure of the spatial distribution pattern was determined using a kriging interpolation method with a spatial interpolation grid.

Statistical analyses were performed using SPSS18.0 (SPSS Inc., IBM Corporation, Chicago, IL, USA) and Excel2007 (Office 2007, Redmond, WA, USA). A semivariogram model, fitted with GS 9.0+ software (Gamma Design Software, GS + 9.0, LLC Plainwell, MI, USA), was used for ordinary kriging interpolation in ArcGIS 9.3 software (ESRI, ArcGIS 9.3, Redlands, CA, USA), rendering a rock exposure spatial distribution map.

## 3. Results and Analysis

### 3.1. Descriptive Statistics for the Coverage of Rock Exposures at Different Sampling Scales

Table 1 provides the statistics on the CRE at different sampling scales. The statistics reveal a wide gap between the maximum and minimum CRE in the Houzhai River basin, which are 95.00% and 0.00%, respectively. The CRE varied slightly with the sampling scale. The 150 m × 150 m scale has the highest average CRE at 15.94%, while the 900 m × 900 m scale had the lowest average CRE at 9.89%. The former is 1.62 times greater than the latter. As the sampling scale increases, the maximum and average values of CRE gradually decrease while the coefficient of variation increases.

The coefficient of variation (CV) is a measure of dispersion of the distribution of a random variable, i.e., the extent of spatial variability in an attribute indicator. Normally, CV values no greater than 10% indicate low variability, CV values between 10% and 100% indicate moderate variability, and CV values greater than 100% signify high variability. The CV in the CRE across the Houzhai River basin ranges from 139.84% to 197.67% (Table 1), suggesting high CRE variability at various sampling scales. This result indicates that the coverage of rock exposures exhibits significant spatial variation within the study area. The average CRE values for the five subsamples fluctuate around the average CRE for each sample set, indicating that the statistical characteristics of the subsamples are consistent with those of the sample and, thus, are representative despite the small number of sampling points in each sample. The CV in CRE first increases and then decreases with increasing sampling scale. Moreover, the CRE is normally distributed in each sample set and all subsamples in terms of both skewness and kurtosis.

### 3.2. Semivariograms Describing the Coverage of Rock Exposures at Different Sampling Scales

The CRE data obtained at different sampling scales were fitted with semivariograms using GS+7.0 Software (Table 2). As seen in Table 2, the CRE values across the study area follow an exponential distribution at different sampling scales. The 150 m × 150 m sample set was used as the reference, and the semivariograms for the five sample subsets were then compared against the semivariogram for the sample set. It was found that the codomain of CRE decreases steadily with increasing sampling scale. This result is possible because a larger sampling scale is associated with a smaller number of samples and thus lower levels of uniformity in the indicators considered. The nugget effect (C_0_) is usually used to measure the variation due to experimental error and negative deviation from actual sampling scale, i.e., spatial heterogeneity caused by random factors. Table 2 shows that the C_0_ peaks in the 150 m × 150 m sample set and declines with increasing sampling scale. This result indicates that the amount of variation caused by random factors tends to decrease as the sample scale increases, possibly because a decrease in sampling scale will increase the number of random factors involved and the complexity of the causes of variability, and thus, more secondary causes will be neglected.

The nugget coefficient of the semivariogram, which is defined as C_0_/C_0_ + C, is 0.512 for the 150 m × 150 m sample set and 0.500 for the 300 m × 300 m sample set. The nugget coefficient is smaller than 0.5 at the scale of 450 m × 450 m, and it begins to decrease as the sampling scale further increases. This result suggests a strong spatial dependence of CRE at sampling scales greater than 450 m × 450 m. This result is possible because the spatial dependence caused by structural and random factors at small scales is covered by that at larger scales. Therefore, an increase in sampling scale can strengthen the effects of structural factors and thereby lead to variability in spatial patterns within a certain region.

### 3.3. Spatial Characteristics of Rocky Desertification in a Small Catchment Area in Karst

Based on the spatial characteristics of the coverage of bedrock exposures in the Houzhai River basin and methods of rocky desertification classification provided by previous research [20], this study classifies the extent of rocky desertification in this area into the following grades: (1) non-karst region (NSD) unaffected by rocky desertification: coverage of bedrock exposures < 20%; (2) potential rocky desertification (PSD): 20% ≤ coverage of bedrock exposures < 30%; (3) light rocky desertification (LSD): 30% ≤ coverage of bedrock exposures < 50%; (4) moderate rocky desertification (MSD): 50% ≤ coverage of bedrock exposures < 70%; and (5) severe rocky desertification (HSD): 70% ≤ coverage of bedrock exposures < 90%. The data from the present study show that at the scale of 150 m × 150 m, slight, moderate, and severe rocky desertification cover areas of 7.81 km^2^, 4.50 km^2^, and 1.87 km^2^, respectively, in the Houzhai River basin.

**Figure 3:**
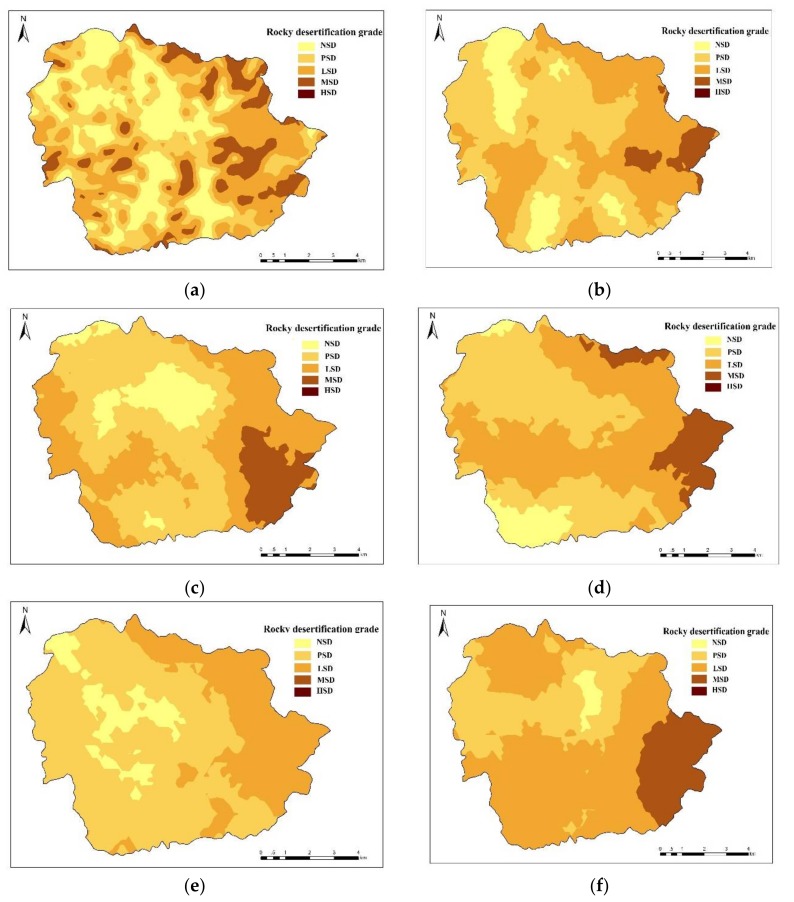
Spatial distribution of rocky desertification under different sampling scales: (**a**) 150 m × 150 m sampling scale; (**b**) 300 m × 300 m sampling scale; (**c**) 450 m × 450 m sampling scale; (**d**) 600 m × 600 m sampling scale; (**e**) 750 m × 750 m sampling scale; (**f**) 900 m × 900 m sampling scale. NSD is non-karst region; PSD is potential rocky desertification; LSD is slight rocky desertification; MSD is moderate rocky desertification; HSD is severe rocky desertification.

Rocky desertification in the study area is distributed primarily in the peak-cluster depressions from the northwest to the southeast. As the sampling scale increases, the distribution of severe rocky desertification varies significantly in the southeastern region, while the distribution of moderate rocky desertification varies little (shown in Figure 3). The northern and central parts of the study area exhibit extensive rocky desertification shrinkage, while the coverage of rocky desertification decreases sporadically in the northern part. The expansion of severe rocky desertification is concentrated in the Yuyangzhai and Dayouzhai villages in the southeast and the Chenqi, Houshan, and Zhaojiatian villages in the north. The trend in the extent of rocky desertification with the number of samples can better characterize the actual evolution of rocky desertification. All variations in the rocky desertification of different grades found in the study area are driven by a combination of natural and human factors. The factors affecting rocky desertification vary with sampling scale. Therefore, determining the different driving factors behind the variations can facilitate more reasonable sampling in research on rocky desertification.

### 3.4. Factors Affecting the Characteristics of Rocky Desertification at Different Sampling Scales

As the grade of rocky desertification depends on the CRE, this study uses the factors affecting the CRE to characterize the factors affecting rocky desertification (Table 3). A Pearson correlation analysis shows that at the scale of 150 m × 150 m, the CRE has extremely significant and positive correlations with slope and elevation (*p* < 0.01), and the correlation coefficients are 0.893 and 0.991, respectively. The correlation with soil thickness is extremely significant and negative (*p* < 0.01), and the correlation coefficient is −0.913. A significantly positive correlation exists between the CRE and slope position (*p* < 0.05), and the correlation coefficient is 0.480. The correlations between the CRE and soil bulk density and rock content were not significant (*p* > 0.05). At the scale of 300 m × 300 m, the CRE has a significantly negative correlation with soil thickness (*r* = −0.732) (*p* < 0.05) and significantly positive correlations with elevation (*r* = 0.512) and rock content (*r* = 0.610) (*p* < 0.05). The correlations with slope (*r* = 0.721) and slope position (*r* = 0.913) are extremely significant and positive (*p* < 0.01). There is no obvious correlation between CRE and soil bulk density. At the scale of 450 m × 450 m, the CRE has extremely significant and positive correlations with slope, elevation, rock content, and slope position (*p* < 0.01, *r* = 0.763, 0.813, 0.913, and 0.680, respectively). At the scale of 600 m × 600 m, the CRE shows an extremely significant and positive correlation with rock content (*p* < 0.01, *r* = 0.684). The CRE has significant, positive correlations with slope and slope position (*p* < 0.05), and the correlation coefficients are 0.503 and 0.406, respectively. There is no significant correlation between the CRE and other factors. At the scale of 750 m × 750 m, the CRE is positively and significantly correlated with rock content and slope position (*p* < 0.05), and the correlation coefficients are 0.780 and 0.741, respectively. At the scale of 900 m × 900 m, the CRE has an extremely significant and negative correlation with soil thickness (*p* < 0.01, *r* = −0.637) and a strong positive correlation with slope position (*p* < 0.05), and the correlation coefficient is 0.501.

The regression equations fitted to the CRE data are shown in Table 4. The values of the coefficient of determination (R^2^) reveal that the distribution of soil bulk density has the poorest fit at all sampling scales, which indicates a relatively weak correlation between soil bulk density and the CRE. As the scale increases, the distributions of topographic factors (elevation and slope) and rock content improve in the goodness of fit to the CRE. Overall, an increase in the sampling scale improves the goodness of fit of the equations. The data obtained at a large scale (e.g., 900 m × 900 m) can describe the relationships between CRE and the factors more accurately, while the data acquired at a small scale (e.g., 150 m × 150 m) is less able to reflect their relationships due to the influence of complex microtopography. Therefore, decreasing the sampling scale may decrease the goodness of fit between the CRE and various influencing factors.

It is clear from the aforementioned findings that the key driving factors behind the spatial variability in the CRE differ depending on the sampling scale. At small scales (150 m × 150 m, 300 m × 300 m and 450 m × 450 m), the spatial variations in the CRE are affected by a combination of slope, elevation, soil thickness, and the CRE. At a medium scale (600 m × 600 m), the spatial variations in the CRE depend on slope, rock content, and CRE. At large scales (750 m × 750 m and 900 m × 900 m), soil thickness and the CRE are the key factors influencing the variability in the CRE. As the sampling scale increases, the structural features attributed to the concentration of multiple complex factors over short distances are hidden by the factors that affect CRE over longer distances, such as soil thickness and the CRE. This result explains why the topographic factors that have relatively stable and continuous distributions (soil thickness and the CRE) show stronger correlations with the CRE at large scales.

## 4. Discussion

### 4.1. Relations between Sampling Scale and Stony Desertification

In general, there are two kinds of understandings about study scales: one is to conduct studies at multiple scales within a fixed study area by encrypting or broadening the sample number, and the other is to conduct studies at multiple scales by changing the study areas from small to large. The two methods reveal different factors or processes and have different characteristics [21]. In some studies on the effects of soil attributes at multiple scales the first method has been utilized. However, the majority of researchers have utilized the second method. They study the sampling scale affect by expanding the study area. This method put emphasis on deduction based on results of studies at different area, and tries to reveal the relationship between sampling scale and study area [22,23,24]. The second method cannot meet the demands of multiscale studies within a fixed area to reflect the global scope. Then, a scale can only reflect the information of local soil characteristics within the sampling range, and it is unable to describe global soil characteristics at different sampling scales [25]. However, it is inevitable that changes to scale will lead to fluctuations in variability, resulting in deviations between apparent variations and real variations [26]. In particular, the karst areas cannot reflect the factors that influence stony desertification areas very well [27]. In view of this, small watersheds were taken as the objects and the study were carried out in an approximately 75 km^2^ area in this study. The first method can more effectively reflect soil information on different scales within the entire basin. With a comprehensive grid sampling method being used in the basin, a comparison was made among different sampling scales that are evenly distributed throughout the basin, which effectively avoids the overgeneralization of the results from studies at small and medium scales.

Karst stony desertification is one of the main types of land desertification [28]. Karst stony desertification occurs in fragile ecological environments and is driven by extremely unreasonable human activities [29,30]. The degradation of land productivity is the essence and the appearance of similar desert landscapes is the symbol of stony desertification [31]. The interference of unreasonable human activities exacerbates the evolution and progress of landscape fragmentation in karst landscapes that are characterized by stony desertification. Therefore, accurately reflecting the spatial distribution patterns of stony desertification areas plays an important role in research on stony desertification [32]. To better describe the spatial variability, it is of practical significance to accurately visualize the relations between spatial distribution and sampling scale in stony desertification areas [33]. The spatial distribution characteristics of stony desertification in the Houzhai River Basin are closely related to the topography and geomorphology of the watershed, of which there are mainly peaks and low-lying lands in the east, mountains in the north, south and southwest, and mainly plains dotted with a few hills in the middle and west. Corresponding to the topographical and geomorphologic features, stony desertification in this watershed is mainly concentrated in the peaks and low-lying lands in the east, the mountainous areas in the north, south and southwest, as well as the buttes in the middle and west.

### 4.2. Influencing Factors of Stony Desertification at Different Sampling Scales

The results show that with the increase in the study scale, the spatial correlation of rock exposure changes from moderate to strong; at the same time, in studies at large scales, the correlations between the gradient, elevation and gradient position and the rock exposure are weakened, and rock contents and soil thickness become the key factors influencing stony desertification in studies at large scales. This conclusion has been widely recognized by other experts, and Wang Dian Jie et al. also believe that different topographic factors act on different scales and the impacts of gradient and elevation are mainly manifested at small and medium studies [34]. With the increase in the study scale, the correlation between rock contents and soil properties is more significant. Chen Shengzi et al. believe that the scale variance at large scales increases with fluctuations as the scale increases, and at this moment, stochastic effects have no obvious influences on soil properties [35].

The gradient and rock exposure of Houzhai River Basin increase with the rise in elevation, while the soil thickness decreases accordingly. It is well known that geographical and climatic conditions at high elevations are poor and not usually suitable for plant growth. However, due to the lack of arable lands, soil at high elevations in the basin is still used for food production, which eventually aggravates the evolution of stony desertification in karst areas. Through a comprehensive analysis, it was found that the gradient, rock exposure, and soil thickness were the main factors that determined the degree of stony desertification [36]. Stony desertification is a serious problem in the Houzhai River Basin. Gradient and elevation are important factors that lead to soil erosion and stony desertification. The larger the gradient, the more serious the soil erosion caused by overland runoff, thus resulting in stony desertification. As the elevation increases, the environmental conditions become worse, including increases in the gradient and reductions in soil thickness. The vegetation condition becomes worse, and the soil and water conservation capacities at high elevations are relatively weak. In addition, in this study, it is also discovered that the key factors influencing the spatial variability of rock exposure vary with sampling scale. At small scales, the rock exposure is influenced by many factors; at medium scales, it is influenced by gradient, rock contents and rock exposure; and at large scales, the soil thickness and rock exposure are the key factors that influence the spatial variability of rock exposure.

## 5. Conclusions

In this paper, there were some differences in assessment of rock exposure at different scales. The average rock exposure is 15.94% at the scale of 150 m × 150 m, which is the largest, and the average rock exposure is 9.89% at the scale of 900 m × 900 m, which is the smallest. With the increase in sampling scale, the maximum value and the average value of rock exposure decreased gradually while the coefficient of variability increased. At the scale of 150 m × 150 m, the areas of minor stony desertification, medium stony desertification and major stony desertification in the Houzhai River Basin are 7.81 km^2^, 4.50 km^2^, and 1.87 km^2^, respectively. The key factors influencing the spatial variability of rock exposure vary with sampling scale. At small scales (150 m × 150 m, 300 m × 300 m, and 450 m × 450 m), the spatial variability of rock exposure is influenced by gradient, elevation, and soil thickness; at medium scales (600 m × 600 m), it is impacted by gradient and soil gravel content; and at large scales (750 m × 750 m and 900 m × 900 m), soil thickness is the key factor influencing the variability of rock exposure. There is about 22.0 million km^2^ of earth surface belonging to the karst landform. This study will present a scientific reference in accurate determination of sampling scale in karst areas.

## Figures and Tables

**Table 1:** Description and statistics of rock exposures under different sampling scales.

Sampling Scales	Sample Size	Minimum	Maximum	Mean	Standard Deviation	Coefficient of Variation	Skewness	Kurtosis
150 m × 150 m	2755	0.00	95.00	15.94	22.29	139.84	1.33	0.79
300 m × 300 m	802	0.00	92.00	12.89	21.42	166.18	1.90	1.69
450 m × 450 m	357	0.00	88.00	12.18	20.71	170.03	1.35	0.78
600 m × 600 m	200	0.00	85.00	11.97	20.51	171.35	1.99	1.71
750 m × 750 m	128	0.00	75.00	10.22	17.52	171.43	2.29	1.72
900 m × 900 m	91	0.00	73.00	9.89	19.55	197.67	2.52	1.83

**Table 2:** Semi-variance model of rock exposures and its fitting parameters under different sampling scales.

Sampling Scales	Model Type	Nugget (C_0_)	Sill (C_0_ + C)	Range/m	C_0_/C_0_ + C	R^2^	RMSE
150 m × 150 m	Index	0.088	0.172	2960	0.512	0.848	0.673
300 m × 300 m	Index	0.076	0.152	2350	0.500	0.831	0.612
450 m × 450 m	Index	0.068	0.272	2630	0.250	0.772	0.478
600 m × 600 m	Index	0.062	0.243	2140	0.255	0.636	0.281
750 m × 750 m	Index	0.043	0.196	1970	0.219	0.779	0.222
900 m × 900 m	Index	0.041	0.187	1860	0.219	0.782	0.228

**Table 3:** Correlation matrix of rock exposures and its influencing factors under different sampling scales.

Sampling Scale	Slope	Elevation	Soil Thickness	Soil Bulk Density	Rock Content	Slope Position
150 m × 150 m	0.893 **	0.991 **	−0.913 **	−0.862	0.510	0.480 *
300 m × 300 m	0.721 **	0.512 *	−0.732 *	−0.602	0.610 *	0.913 **
450 m × 450 m	0.763 **	0.813 **	0.416	0.402	0.913 **	0.680 **
600 m × 600 m	0.503 *	0.453	0.436	0.436	0.684 **	0.406 *
750 m × 750 m	0.421	0.112	0.462	0.302	0.780 *	0.741 *
900 m × 900 m	0.689	0.363	−0.637 **	0.412	0.496	0.501*

** Indicates that the correlation is significant when the confidence level (double measure) is 0.01; * the correlation is significant when the confidence level (double measure) is 0.05.

**Table 4:** Optimal fitted equations of factors influencing rock exposures at different sampling scales.

Sampling Scale	Index	The Optimal Fitted Equation for Rock Exposures	R^2^
150 m × 150 m	Slope	*y* = 0.0112*x* + 12.539	0.263
	Elevation	*y* = 0.166*x* + 1288.201	0.287
	Soil thickness	*y* = 0.0212*x* + 11.592	0.272
	Soil bulk density	*y* = −0.0255Ln(*x*) + 1.1502	0.011
	Slope position	*y* = 0.1*x*^2^ + 0.031*x* + 9.358	0.382
	Rock content	*y* = 1.3256Ln(*x*) − 1.4693	0.369
300 m × 300 m	Slope	*y* = 0.0194*x* + 22.384	0.343
	Altitude	*y* = 0.0194*x* + 1425.084	0.298
	Soil thickness	*y* = 0.0194*x* + 15.084	0.349
	Soil bulk density	*y* = 1.2081Ln(*x*) + 11.31	0.017
	Slope position	*y* = 0.2*x*^2^ + 0.0048*x* + 5.695	0.439
	Rock content	*y* = 1.2682Ln(*x*) − 12.225	0.428
450 m × 450 m	Slope	*y* = 0.0231*x* + 10.148	0.391
	Altitude	*y* = −2.3608*x* + 1314.8	0.317
	Soil thickness	*y* = −0.0216*x* + 17.978	0.361
	Soil bulk density	*y* = 1.3498Ln(*x*) + 12.466	0.018
	Slope position	*y* = 0.2*x*^2^ − 0.1014*x* + 0.2054	0.463
	Rock content	*y* = −3.2414Ln(*x*) + 21.321	0.474
600 m × 600 m	Slope	*y* = 0.0182*x* + 24.713	0.412
	Altitude	*y* = 0.131*x* + 1001.571	0.331
	Soil thickness	*y* = 43.525 − 0.950*x* + 0.105*x*^2^	0.372
	Soil bulk density	*y* = 1.2014Ln(*x*) + 13.823	0.020
	Slope position	*y* = 0.0003*x*^2^ + 0.0681*x* + 15.567	0.514
	Rock content	*y* = 1.0924Ln(*x*) + 21.854	0.548
750 m × 750 m	Slope	*y* = 0.1313*x* + 23.153	0.497
	Altitude	*y* = 0.101*x* + 1033.201	0.395
	Soil thickness	*y* = −0.1625*x* + 65.293	0.619
	Soil bulk density	*y* = 3.1781Ln(*x*) + 15.597	0.022
	Slope position	*y* = 0.002*x*^2^ − 0.0855*x* + 16.946	0.587
	Rock content	*y* = 0.1313*x* + 22.053	0.649
900 m × 900 m	Slope	*y* = 0.2669*x* − 0.0397	0.516
	Altitude	*y* = 0.136*x* + 1083.201	0.491
	Soil thickness	*y* = 0.1325*x* + 24.78	0.673
	Soil bulk density	*y* = 0.9136Ln(*x*) + 21.2	0.029
	Slope position	*y* = 0.0023*x*^2^ − 0.1766*x* + 17.997	0.645
	Rock content	*y* = 0.8333Ln(*x*) + 21.429	0.703
